# The Impact of Age on Mortality and Disability in Patients With Ischemic Stroke Who Underwent Cerebral Reperfusion Therapy: A Brazilian Cohort Study

**DOI:** 10.3389/fnagi.2021.649902

**Published:** 2021-07-06

**Authors:** Natália Eduarda Furlan, Gustavo José Luvizutto, Pedro Tadao Hamamoto Filho, Silméia Garcia Zanati Bazan, Gabriel Pinheiro Modolo, Natalia Cristina Ferreira, Luana Aparecida Miranda, Juli Thomaz de Souza, Fernanda Cristina Winckler, Edison Iglesias de Oliveira Vidal, Carlos Clayton Macedo de Freitas, Luis Cuadrado Martin, Rodrigo Bazan

**Affiliations:** ^1^Faculdade de Medicina de Botucatu, Universidade Estadual Paulista Júlio de Mesquita Filho (UNESP), Botucatu, Brazil; ^2^Departamento de Fisioterapia Aplicada, Universidade Federal do Triângulo Mineiro, Uberaba, Brazil

**Keywords:** stroke, cerebral reperfusion, rtPA, thrombectomy, elderly

## Abstract

**Introduction:** The main driver for increased stroke prevalence is the aging of the population; however, the best evidenced-based strategies for stroke treatment and prevention are not always followed for older patients. Therefore, the aim was studying the association of age with clinical outcomes (mortality and functional disability) in stroke patients who underwent cerebral reperfusion therapy at hospital discharge and 90 days after ictus.

**Methods:** This was a retrospective (stroke databank analysis) cohort study of participants who had been diagnosed with ischemic stroke and undergone intravenous cerebral reperfusion therapy or mechanical thrombectomy. The variable of interest was patient age, which was categorized into four groups: (1) up to 59 years; (2) 60 to 69 years; (3) 70 to 79 years old; and (4) above 79 years. The primary outcome was mortality at hospital discharge and 90 days after stroke, and the secondary outcome was functional capacity at hospital discharge and 90 days after stroke.

**Results:** A total of 281 patients was included in the study (235 treated by thrombolysis alone, and 46 treated with mechanical thrombectomy). The mean age of the total sample was 67 ± 13.1 years. The oldest patients had the most unfavorable outcomes, except for mortality rate, at hospital discharge (mRS > 2; OR: 1.028; 95% CI 1.005 to 1.051; *p* = 0.017; mRS > 3; OR: 1.043, 95% CI 1.018 to 1.069; *p* = 0.001) and 90 days after stroke (mRS > 2; OR: 1.028; 95% CI 1.005 to 1.051; *p* = 0.017; mRS > 3; OR: 1.043, 95% CI 1.018 to 1.069; *p* = 0.001).

**Conclusion:** Cerebral reperfusion was a viable treatment for ischemic stroke in both elderly and very elderly patients, as it did not increase mortality. However, it was observed that older individuals had worse functional outcomes at hospital discharge and 90 days after stroke.

## Introduction

Age is the main non-modifiable risk factor for stroke (Boehme et al., 2017; Sharrief and Grotta, 2019). With the aging of the world population and the Latin American population, there has been an increase in chronic diseases, including stroke, which causes morbidity, mortality, and disability (Bonita, 1992; Zhao et al., 2018). Elderly stroke patients have higher mortality and morbidity and worse functional recovery than younger patients (Porcello Marrone et al., 2013).

Ischemic stroke is responsible for 80% of all cases (Donkor, 2018). In this condition, there is a neurological deficit caused by obstruction of arterial circulation due to a thrombus or embolus that causes hypoxia and hypoglycemia, which leads to infarction of brain tissues (Doyle et al., 2008). Early recanalization due to thrombolysis or thrombectomy associated with care in stroke units is one of the main treatment strategies in the acute phase of stroke; the faster and more effective cerebral reperfusion is, the greater the chance of a good functional outcome (Hacke et al., 2004; IMS Study Investigators, 2004).

Studies revealed that stroke mostly affects older people, and in Brazil, high stroke rates occur in individuals older than 70 years (Bensenor et al., 2015). Most of the epidemiological indicators (incidence, prevalence, mortality-to-incidence ratio, mortality, disability-adjusted life years, years lost due to disability, and years of life lost) of stroke in general or for either type of stroke were higher in elderly individuals (de Santana et al., 2018).

Previous epidemiological studies on the extent of the interaction and influence of stroke severity on clinical outcomes were performed, and the authors showed that baseline stroke severity, along with age, is a major determinant of post-stroke outcome (Bhaskar et al., 2017). In addition, other studies highlighted that elderly patient suffer a disproportionate burden of stroke and are at high risk of poor outcomes (Fonarow et al., 2010). The literature suggests that with increasing age, evidence-based guidelines for stroke care are less likely to be followed (Sharrief and Grotta, 2019). Treatment rates improved substantially over time for ischemic stroke patients in all age groups, and many cohorts describe the favorable results of treatment by thrombolysis or mechanical thrombectomy worldwide (Hacke et al., 1998; Fonarow et al., 2010; Martins et al., 2020), however, reports in elderly patients are less frequent, particularly in Latin American countries.

In low- and middle-income countries, life expectancy is increasing and the population is aging. However, this population usually presents multiple chronic physical diseases, physical and mental health comorbidity and little access to health services (Garin et al., 2016; Rzewuska et al., 2017). Therefore, understanding the behavior of clinical outcome variables in this population in real life and outside of clinical trials, can safely reinforce the inclusion of this population in this type of therapy. Based on this background, the aim was studying the association of age with clinical outcomes (mortality and functional disability) in stroke patients who underwent cerebral reperfusion therapy at hospital discharge and 90 days after ictus. The main hypothesis of this study is that older individuals who have undergone cerebral reperfusion therapy will face no increase in mortality rate but will have worse functional outcomes than individuals who have not undergone this therapy.

## Methods

This study was approved by the Institutional Review Board and followed the recommendations from STROBE (Strengthening the Reporting of Observational studies in Epidemiology) (von Elm et al., 2007).

### Study Design

This is a retrospective cohort study of participants who have been diagnosed with ischemic stroke and undergone intravenous cerebral reperfusion therapy or mechanical thrombectomy.

### Setting

The data collection was carried out at the Botucatu Stroke Unit from June 2012 to September 2020. Botucatu is a city in the southeastern region of Brazil and is located 224.8 km (139.7 mi) from São Paulo, the capital of the state of São Paulo. It has an estimated population of 148,130 (as of 2020) in an area of 1,482.64 km.

### Participants

#### Eligibility Criteria

The study included participants who suffered ischemic stroke that was confirmed by neuroimaging, were > 18 years old, did not have a history of stroke, and had undergone cerebral reperfusion therapy. Participants were excluded if they presented other neurological diseases, hemorrhagic stroke confirmed by CT or MRI scan or stroke mimics.

All patients were admitted to the stroke unit in the first 48 h of the ictus or were referred to the intensive care unit (ICU) in severe cases or in cases of clinical instability that required intensive care support. After admission, patients were followed up at the cerebrovascular disease outpatient clinic at 90 days.

### Variables

#### Independent Variable

The variable of interest was the patient's age, which was categorized into four groups: (1) up to 59 years; (2) from 60 to 69 years; (3) from 70 to 79 years; and (4) above 79 years.

### Outcomes

#### Primary

The primary outcome was mortality at hospital discharge and 90 days after stroke.

#### Secondary

The secondary outcome was functional capacity at hospital discharge and 90 days after stroke.

#### Data Charting Process

Two calibrated physicians extracted data from the included patients. All variables (confounders factors and outcomes) were extracted by stroke data bank of Botucatu Medical School. The database on monthly audit by the stroke unit coordinator.

A standardized data extraction form created by authors was used and the following details were recorded from each patient: (a) confounders factors: sex, ethnicity, stroke severity by the NIHSS scale at admission, presence of previous diabetes and hypertension, smoking, blood pressure value at hospital admission, value of serum creatinine, symptomatic hemorrhagic transformation, time between stroke and arrival at the hospital and type of treatment (thrombolysis alone or associated with mechanical thrombectomy); (b) outcomes: the National Institutes of Health Stroke Scale (NHISS) (Cincura et al., 2009) and modified Rankin scale (mRS) (Cincura et al., 2009) were extracted at the time of hospital discharge and 90 days. Patients were classified according to the modified Rankin scale: >2 (worse outcome) and equal to 6 (death) at two points: at hospital discharge (primary outcome) and at 90 days after discharge (secondary outcome).

### Statistical Analysis

The data are described as the mean ± standard deviation. The normality of continuous variables was assessed by Kolmogorov-Smirnov test. The patients were divided into four age groups, which were compared by analysis of variance (variables with parametric distributions) or the Kruskal-Wallis test (variables with non-parametric distributions) for non-categorical data or by chi-square when appropriate. ROC curves were generated using age as the independent variable and the modified Rankin scale as the outcome (dependent variable) and categorized according to the different cutoff values at hospital discharge and 90 days. The cutoff points were determined by the highest sum of sensitivity and specificity (Youden index).

The association of age with the primary outcome was assessed by binary logistic regression. The odds ratio for this association was adjusted for variables that differed between age groups with *p* < 0.05. Thus, they composed the multiple logistic regression analysis, taking the patient's outcome as a dependent variable and the patient's age as an independent variable. The presence of diabetes, previous mRS score and NIHSS score were included in the model due to their strong association with stroke outcomes. The associations were considered statistically significant if the *p*-value was <0.05. Data analysis was performed using IBM SPSS Statistics® Version 21 software.

## Results

**Figure 1:**
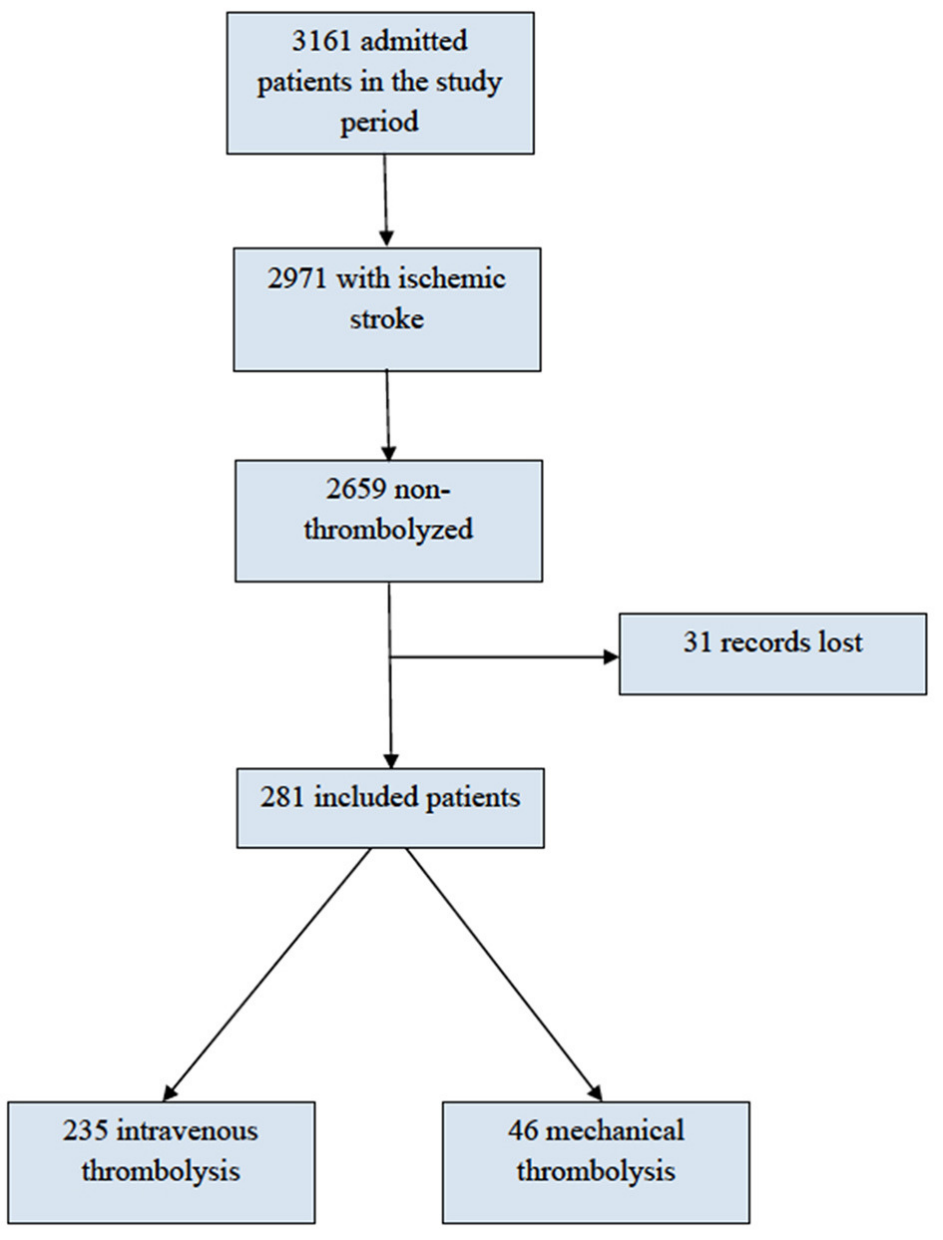
Flow chart.

During the study period, 3,161 patients were admitted to the stroke unit; of these, 2,971 had experienced an ischemic stroke. A total of 2,659 were not subjected to any type of cerebral reperfusion therapy, and 31 patients had lost records, which resulted in 281 patients being included in the study (Figure 1). Of the included patients, 235 underwent treatment by thrombolysis alone, and 46 underwent mechanical thrombectomy (19 mechanical thrombectomy only and 27 mechanical thrombectomy associated with intravenous thrombolysis). The eligibility rate for cerebral reperfusion was 9.5%.

The mean age of the total sample was 67 ± 13.1 years, 89% of the patients were white, and 57% were male; 76% had hypertension, 30% had diabetes, 29% were active smokers, and 19% were previous smokers. Upon admission, the mean NIHSS score was 13 ± 6.9, and the mean values for systolic blood pressure (SBP) and diastolic blood pressure (DBP) were 158 ± 30.5 and 89 ± 17.1 mmHg, respectively. During hospitalization, the mean value of plasma creatinine was 1.1 ± 0.71 mmHg, and 17% presented symptomatic hemorrhagic transformation after treatment. The average mRS at discharge was 3 ± 2.2, and after 90 days, it was 3 ± 2.1. The rate of death within 90 days after stroke was 18% (51 patients), with 14% (40 patients) during hospitalization. The mortality rate according to age group was 13.16% for patients up to 59 years old, 12.34% for patients 60 to 69 years old, 14.06% for patients 70 to 79 years old, and 18.33% for patients above 79 years old.

Table 1 shows the demographic and clinical data according to age groups. There was a statistically significant difference between the age groups regarding the following characteristics: sex, hypertension, smoking, SBP, time between stroke ictus and hospital arrival and frequency of mechanical thrombectomy. Older people had a higher frequency of females, had more hypertension, smoked less, had greater SBP at admission, experienced less time between stroke ictus and hospital arrival, and underwent mechanical thrombectomy less frequently than younger people. The other characteristics studied were homogeneous between groups. The modified Rankin score outcomes at discharge and at 90 days also showed higher values in individuals > 79 years.

**Table 1:** Demographic and clinical data according age group.

	**Age group**				***P***
	** <60**	**60-69**	**70-79**	**>79**	
Age (years)	51 ± 7.3	65 ± 2.8	74 ± 2.6	85 ± 4.0	-
**Females (%)**	**43**	**30**	**41**	**62**	**0.002**
Non-Caucasian (%)	17	9	9	7	0.179
NIHSS	14 ± 7.5	12 ± 6.2	12 ± 6.7	13 ± 7.0	0.270
Diabetes (%)	24	28	36	32	0.442
**Hypertension (%)**	**67**	**73**	**84**	**83**	**0.046**
Smoke (%)					
**Active**	**36**	**36**	**23**	**17**	**0.012**
**Previous**	**25**	**19**	**16**	**13**	
**SBP (mmHg)**	**147** **±** **30.2**	**161** **±** **28.7**	**163** **±** **31**	**163** **±** **29.6**	**0.002**
DBP (mm Hg)	87 ± 19.5	90 ± 15.8	91 ± 16.7	87 ± 15.8	0.366
Creatinine (mg/dL)	1.0 ± 0.56	1.1 ± 0.77	1.2 ± 0.88	1.1 ± 0.38	0.343
Hemorrhagic (%)	25	12	13	17	0.141
**TID (min)**^**^¥^**^	**164** **±** **114.8**	**148** **±** **98.3**	**126** **±** **76.5**	**121** **±** **99.1**	**0.019**
**Thrombectomy (%)**	**21**	**15**	**23**	**5**	**0.025**
mRS^**^¥^**^					
**Discharge**	**2** **±** **1.9**	**2** **±** **2.1**	**3** **±** **2**	**3** **±** **1.9**	**0.003**
**90-days**	**2** **±** **1.9**	**2** **±** **2.1**	**3** **±** **2.1**	**3** **±** **2.0**	**0.002**

*NIHSS, National Institute of Health Stroke Scale; SBP, systolic blood pressure; DBP, diastolic blood pressure; mRS, modified Rankin scale; TID, time ictus-door*;

¥ *Non-parametric test*.

**Figure 2:**
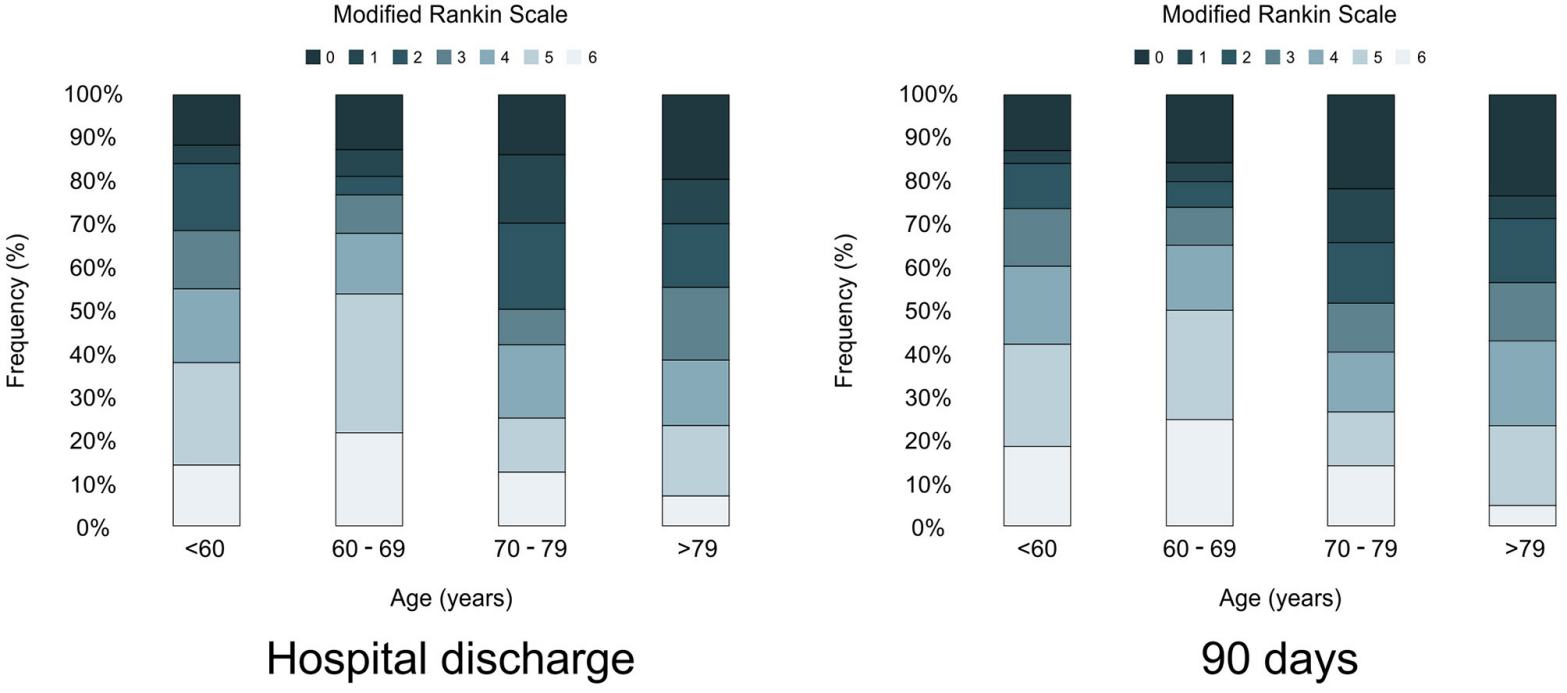
Frequency of modified Rankin scale in each aging group at hospital discharge and 90-days after stroke.

Figure 2 shows the distribution of the mRS according to the age group at hospital discharge and 90 days after stroke. In general, there was an increase in the frequency of higher mRS values among the older age groups but no increase in the mortality rate.

**Figure 3:**
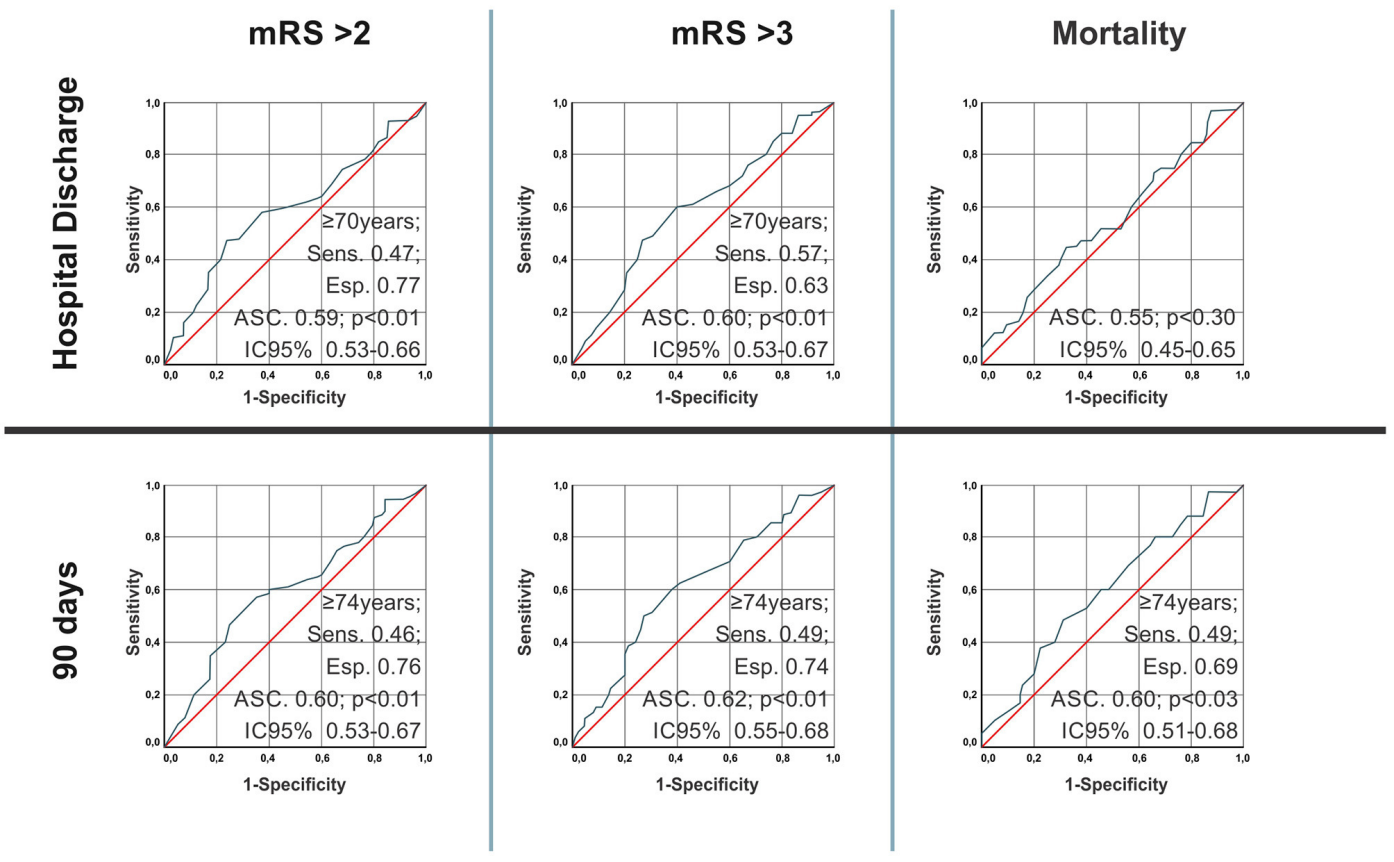
Age discriminatory power for the outcomes of mRS > 2, mRS > 3, and death (mRS = 6) at hospital discharge and after 90 days.

Figure 3 shows the age discriminatory power for the outcomes of mRS > 2, mRS > 3 and death (mRS = 6) at hospital discharge and after 90 days. Age presented statistically significant discriminatory power for the disability outcomes both at hospital discharge and after 90 days. Age predicted death only in 90 days. In general, the age of 74 years presented the best cutoff point, with a low sensitivity (patients with the outcome were below that age) but high specificity (among the patients who presented this outcome, most were above that age).

When adjusting the age odds ratio in years for the association with mortality and functional disability at discharge and 90 days after stroke, for the confounding variables sex, previous mRS score, hypertension, diabetes, smoking, mechanical thrombectomy, onset stroke time to hospital arrival and NIHSS score (Table 2), the oldest patients were those with the most unfavorable outcomes, except for mortality rate, at hospital discharge (mRS > 2; OR: 1.028; 95% CI 1.005 to 1.051; *p* = 0.017; mRS > 3; OR: 1.043, 95% CI 1.018 to 1.069; *p* = 0.001) and 90 days after stroke (mRS > 2; OR: 1.028; 95% CI 1.005 to 1.051; *p* = 0.017; mRS > 3; OR: 1.043, 95% CI 1.018 to 1.069; *p* = 0.001).

**Table 2:** Analysis of binary logistic regression considering age in years as the variable of interest and mRS > 2, mRS > 3, and death (mRS = 6) at hospital discharge and 90 days after stroke as the outcome variables.

**mRS**	**OR**		**CI 95%**		**P**
			**Inferior**	**Superior**	
Hospital discharge					
	**>2**	**1.028**	**1.005**	**1.051**	**0.017**
	**>3**	**1.043**	**1.018**	**1.069**	**0.001**
	Death	1.016	0.983	1.050	0.340
90 days					
	**>2**	**1.028**	**1.005**	**1.051**	**0.017**
	**>3**	**1.043**	**1.018**	**1.069**	**0.001**
	Death	1.030	0.999	1.061	0.055

*OR, odds ratio adjusted by sex, hypertension, diabetes, time ictus-door, prior mRS, NIHSS, and thrombectomy*.

## Discussion

Older people had more hypertension, smoked less, had a greater SBP at admission, experienced less time between stroke ictus and hospital arrival and underwent mechanical thrombectomy less frequently than younger individuals. In the adjusted analysis, those in the older age groups had higher mRS scores and worse outcomes than the other age groups, with 74 years being the best cutoff, except for the mortality rate.

Fast and adequate treatment in the acute phase of stroke reduces complications, disability and death in the long term (Martins et al., 2007). Elderly individuals are more likely to have stroke with greater neurological severity (de Carvalho et al., 2011). de Santana et al. (2018) observed high disability, mainly in individuals above 70 years (de Santana et al., 2018). In this study, we observed high mRS scores at hospital discharge and 90 days after stroke in elderly individuals, although there was no increase in mortality rate between age groups.

Nagajara et al. (2021) showed that oldest old patients who received t-PA alone or MT alone had remarkably worse outcomes for in-hospital mortality and discharge to home than young adults. However, in a systematic review, the authors showed that participants aged over 80 years benefited equally from those aged under 80 years, particularly if treated within 3 h from stroke onset (IST-3 collaborative group et al., 2012; Wardlaw et al., 2014), and Ahmed et al. (2017) concluded that elderly patients should not be denied thrombolysis solely on the basis of age.

However, the rate of thrombolysis in this study was ~10%, which is a low rate compared to that found in American and European cohorts but is greater than that in other Latin American cohorts due to a lack of public health organizations and campaigns to improve public education (Abanto et al., 2020). In addition, only 5% of patients aged 80 and over underwent mechanical thrombectomy. These factors may also have been determinants for worsening the outcome in elderly individuals.

Some conditions could be associated with worse outcomes in elderly stroke patients. Some authors highlight that the patients who arrive at the hospital earlier are the most severe, and due to their alarming clinical condition, they tend to have access to the hospital service faster (Valiente et al., 2008). In this cohort, elderly individuals had less time between the onset of stroke symptoms and arrival at the hospital, which may indicate greater clinical severity.

Another important factor was that 76% of the individuals had hypertension and greater SBP at hospital admission, demonstrating the high prevalence of this main risk factor in the studied population (Panicio et al., 2014). The brain is one of the main target organs affected by hypertension (Dahlöf et al., 2002; Faraco and Iadecola, 2013). High blood pressure (BP) is associated with worse clinical outcomes in the setting of acute ischemic stroke (Iadecola et al., 2016). Emerging evidence suggests that hypertension may also play an important role in the development of cognitive decline and vascular dementia after stroke (Maïer et al., 2017), and the favorable outcome rate was the highest at low SBP values and lowest at high SBP values in the acute phase of stroke (Walker et al., 2017).

In addition to the above-mentioned conditions, chronic diseases had a strong influence on the functional capacity of elderly individuals (Alves et al., 2010). We must highlight that the aging of the Brazilian population is different when compared to the population of high-income country, because the elderly has a high number of comorbidities (risk factors) and have difficulty controlling risk factors at the optimal level.

We acknowledge limitations to our study. First, this is a unicentric and retrospective cohort study based on electronic medical charts. Second, the ASPECTS was not scored at admission, and no modality of neuroimaging was performed to verify the recanalization rate. Third, we did not have a cohort of non-thrombolyzed patients for comparison between groups; finally, frailty was not evaluated in the elderly patients, and this may have been an important contributor to the oldest old patients having worse outcomes. Despite these limitations, electronic medical records were a good source of information that allowed for adequate data retrieval in 90% of patients. On the other hand, it is noteworthy that only stroke patients treated with some type of thrombolysis were studied, which is less frequent in the literature, particularly among Latin Americans.

In recent years, treatment with cerebral reperfusion in acute stroke has been the gold standard treatment in some cases. Regarding the risk and benefit balance between elderly and very elderly people with stroke, in relation to cerebral reperfusion, this issue has not yet been fully resolved. The data in the present study show a less favorable evolution among elderly people undergoing reperfusion but no increase in mortality rates.

Sobolewski et al. (2020) suggested that patients aged ≥80 may be safely treated with cerebral reperfusion in routine practice. The results of this Latin American cohort study also reaffirm the benefits of thrombolytic therapy for stroke patients in this age group. Age is an independent risk factor for disabilities; however, the mortality rate of the elderly patients was more related to comorbidities and not to age *per se*. Cerebral reperfusion in elderly stroke patients proved to be safe in this study. This work may encourage stroke neurologist to include these patients in future clinical trials about mechanical thrombectomy. However, the indication criteria should be evaluated more rigorously in very elderly patients due to the limited facilities of care in elderly stroke.

## Conclusion

Cerebral reperfusion was a viable treatment for ischemic stroke in both elderly and very elderly patients, as it did not increase mortality. However, it was observed that older individuals had worse functional outcomes at hospital discharge and 90 days after stroke.

## Data Availability Statement

The raw data supporting the conclusions of this article will be made available by the authors, without undue reservation.

## Ethics Statement

The studies involving human participants were reviewed and approved by Botucatu Medical School. The patients/participants provided their written informed consent to participate in this study.

## Author Contributions

All authors listed have made a substantial, direct and intellectual contribution to the work, and approved it for publication.

## Conflict of Interest

The authors declare that the research was conducted in the absence of any commercial or financial relationships that could be construed as a potential conflict of interest.
